# Owners of a conspiratorial heart? Investigating the longitudinal relationship between loneliness and conspiracy beliefs

**DOI:** 10.1111/bjso.12865

**Published:** 2025-02-20

**Authors:** Tisa Bertlich, Anne‐Kathrin Bräscher, Sylvan Germer, Michael Witthöft, Roland Imhoff

**Affiliations:** ^1^ Johannes Gutenberg University Mainz Mainz Germany

**Keywords:** conspiracy mentality, COVID‐19 conspiracy beliefs, loneliness, longitudinal, random intercept cross‐lagged panel model

## Abstract

Feeling positively connected to other people is a basic human need. If this need is threatened by feeling lonely, people might become more susceptible to conspiracy theories to help make sense of their surroundings. Simultaneously, conspiracy beliefs could lead to loneliness because they can strain existing relationships. Using two pre‐registered longitudinal studies, we investigated the reciprocal relationship between loneliness and conspiracy mentality (Study 1, *N* = 1604) and the more malleable specific conspiracy beliefs (Study 2, *N* = 1502) during the COVID‐19 pandemic. Random‐intercept cross‐lagged panel models showed that people who are, on average, lonelier are also more likely to believe in conspiracy theories. However, the data provided no support for the notion that conspiracy beliefs and loneliness predicted each other over time. The research helps to understand the thus far mixed evidence on loneliness and conspiracy beliefs and adds important insights to the literature on conspiracy beliefs and need deprivation.

## INTRODUCTION

During the Covid‐19 pandemic, societies and individuals were faced with a variety of challenges. In the beginning, it was unclear where the virus came from, how it spreads, and how severe it is. Around these questions, conspiracy theories arose. Believing in such theories has been linked to numerous negative consequences such as prepping behaviour (Imhoff & Lamberty, 2020), risky health choices (Bierwiaczonek et al., 2022), and loneliness (Hettich et al., 2022). Loneliness itself has been pointed out as an important challenge of the pandemic, too (Armitage & Nellums, 2020; Banerjee & Rai, 2020). It describes the painful feeling of not having the quality or quantity of social relationships one desires (Perlman & Peplau, 1981). Due to preventive measures, people were unable to pursue their pre‐pandemic hobbies, go to work, or see their loved ones. This decreased social interaction is a risk factor for loneliness (Dahlberg et al., 2022). Indeed, a meta‐analysis of high‐quality studies shows that levels of loneliness increased during the pandemic (Ernst et al., 2022). As conspiracy beliefs and loneliness changed dynamically during the pandemic, the study at hand investigates whether and how these two factors are related.

A conspiracy theory can be defined as the explanation of an event or phenomenon with a secret plot by powerful people. If a person believes in one or more of such explanations, we speak of conspiracy beliefs. It is a well‐established finding that believing in one conspiracy theory increases the likelihood that a person will believe in another, unrelated conspiracy theory (Goertzel, 1994; Wood et al., 2012). While some argue that this indicates a network of reinforcing beliefs (Williams et al., 2022), others see this as an indication of a conspiracy mentality, which captures the rather stable observation that people tend to differ in their tendency to see evil plots at play in society (Imhoff & Bruder, 2014). Despite this intimate relation between specific conspiracy beliefs and a more generalised conspiracy mentality, the former have generally been more susceptible to experimental manipulations and hence less stable over time (Imhoff et al., 2022). However, both specific conspiracy beliefs and conspiracy mentality have been linked to feelings of loneliness (Hettich et al., 2022; Jolley et al., 2023; Neu et al., 2023).

### Evidence for the link between conspiracy beliefs and loneliness

Numerous related but distinct individual‐level characteristics point to a relationship between conspiracy beliefs and loneliness: People who feel alienated from the political system, people with lower interpersonal trust, and those who feel like the social fabric of society is breaking down are more likely to believe in conspiracy theories (Abalakina‐Paap et al., 1999; Goertzel, 1994; Imhoff & Lamberty, 2018; Meuer & Imhoff, 2021). Specifically looking at loneliness, real‐life data from conspiracy forums show that social isolation and loneliness are explicit topics within some conspiracy communities (Jeltsen, 2018; Maxwell et al., 2020). For example, a thematic content analysis investigating the themes within the *Incel* (*In*voluntary *Cel*ibacy) community showed that many self‐identified Incels report high levels of loneliness and social isolation and that many lack deep social connections (Maxwell et al., 2020). Similarly, participants in an interview study with 15 former conspiracy believers reported that both initial engagement with conspiracy theories and leaving conspiracy communities come with the cost of increased perceived social isolation and thus loneliness (Engel et al., 2023). Quantitative studies point in a similar direction: A series of partly large‐scale and cross‐sectional studies find a small positive association between both conspiracy mentality/specific conspiracy beliefs and loneliness (.10 < *r* < .28; Alsuhibani et al., 2022; Hettich et al., 2022; Jolley et al., 2023; Neu et al., 2023).

Experimental evidence investigating the causal relationship between conspiracy beliefs and loneliness is lacking thus far. This may be partially attributable to the fact that neither lends itself to an easy experimental manipulation. Experimentally manipulating conspiracy beliefs is difficult and plagued with unreliable findings (Imhoff et al., 2022). A recent experimental study randomly assigned participants to remember a lonely time in their life vs. a control condition, which increased reports of feeling lonely (Jolley et al., 2023). The fact that participants in the lonely condition did not show a greater belief in conspiracy theories, however, cannot be taken as strong evidence against the causal pathway between loneliness and conspiracy beliefs. As the authors discuss, this lack of an effect could be due to the manipulation being too weak or that loneliness must be experienced for a longer period to increase the need to get access to new communities, which could not be captured in this kind of short‐term experimental design.

Regardless, there is reason to assume that loneliness and conspiracy beliefs are causally linked. In the next section, we will first address the idea that loneliness increases conspiracy beliefs before turning to the other causal direction.

### Loneliness predicting conspiracy beliefs

Most psychologists agree that conspiracy beliefs owe part of their lure to the fact that they promise to satisfy important psychological needs (Douglas et al., 2017). Therefore, loneliness could increase conspiracy beliefs because it signals that two types of needs are under threat: the social need to belong and the existential need for safety and control.

First, loneliness is an unpleasant feeling that signals lack of companionship and motivates the lonely person to reduce it by seeking new social relationships or repairing existing ones (Cacioppo et al., 2013). Conspiracy theories might promise to alleviate this unpleasant state by offering access to a community of like‐minded people (Biddlestone et al., 2025; Klein et al., 2019). Conspiracy theories could be particularly alluring for individuals seeking connection online because social media algorithms are more likely to favour such often emotion‐provoking content (Imhoff, 2023). This idea gains indirect support from an interview study with conspiracy believers, in which some of them expressed that conspiracy groups gave them a sense of community (Franks et al., 2017).

Second, loneliness increases existential threat, which can foster conspiracy beliefs. Loneliness indicates that an individual is on their own, without allies to protect each other. At the same time, loneliness is thought to signal that those around you are not friends but another potential source of threat (Cacioppo et al., 2015; Qualter et al., 2015). Increased threat, again, could make people more susceptible towards conspiracy theories because they provide the individual with a coherent and straightforward explanation for the environment and thereby the cause of the threat (van Prooijen, 2019). Both arguments above receive indirect support through research that shows that experimentally inducing ostracism increased the endorsement of conspiracy theories (Poon et al., 2020). Current research assumes that ostracism deprives a whole range of basic needs (Williams, 2009), among them the *social* need to belong and the *existential* need for control.

Even though this need deprivation lens is very popular among conspiracy researchers, experimental evidence for the causal link between deprived needs and conspiracy beliefs is still scarce and mixed (see Biddlestone et al., 2025; Stojanov & Halberstadt, 2020). First longitudinal research even points in the opposite causal direction (Liekefett et al., 2021). Therefore, it is important to also consider the causal pathway from conspiracy beliefs to loneliness.

### Conspiracy beliefs predicting loneliness

There is also reason to believe that conspiracy beliefs increase feelings of loneliness. First, conspiracy beliefs could lead to attitudinal distancing (Toribio‐Flórez et al., 2023). Taking up conspiracy beliefs could change attitudes regarding the object of the conspiracy, which could lead to conspiracy believers suddenly experiencing greater attitudinal distance from the people around them. This, again, could decrease interpersonal liking. This attitudinal distancing could also lead to behavioural changes: conspiracy believers might overstep social norms, possibly because of their own skewed perception of social norms (Pummerer, 2022). Violating these norms could deteriorate social trust and interpersonal relationships (Toribio‐Flórez et al., 2023).

First studies support the idea that believing in conspiracy theories strains existing relationships, especially with people who do not believe in conspiracy theories. For example, research on real‐life relationships with people who have family members or friends who believe in QAnon conspiracy theories showed that participants report a decreased relationship quality since the friend or family member started believing in QAnon (Moskalenko et al., 2022; Mousaw, 2022). Experimental studies point in a similar direction: In a series of studies, participants were instructed to name a person from their social network and then asked to either imagine that this person believed in conspiracy theories (conspiracy condition) or not (control condition). People in the conspiracy condition reported lower relationship satisfaction, particularly those not believing in conspiracy theories themselves (Toribio‐Flórez et al., 2024).

Second, it may be more difficult for conspiracy believers to form new social relationships because conspiracy beliefs can be stigmatising. The label “conspiracy theory” is often used to refer to implausible, deviant beliefs (Barkun, 2015) that are used to discredit a claim (Douglas et al., 2021). Therefore, conspiracy beliefs might have a negative impact on the impression formation of people, making it more difficult to form positive social bonds between conspiracy believers and non‐believers (Green et al., 2023; Toribio‐Flórez et al., 2023).

This is supported by a study investigating conspiracy beliefs around a plane crash in which many representatives of the Polish government died in 2010: In this study, non‐conspiracy believers preferred more physical and relational distance from conspiracy believers than the other way around (Bilewicz et al., 2019). Experimental evidence also shows that participants who were instructed to write a text of three arguments in favour of (or opposing) a popular conspiracy theory with the goal of convincing readers anticipated more negative evaluation from the audience and were more concerned about being socially excluded, a potential cause of loneliness (Lantian et al., 2018).

### The present research

Taken together, a considerable amount of research points to an association between conspiracy beliefs and loneliness. However, it is still unknown whether they are causally linked and if so, how. It is plausible that loneliness increases the vulnerability for conspiracy beliefs and vice versa. In the present research, we thus try to approximate this question with two large‐scale longitudinal studies taking place during the COVID‐19 pandemic.

In Study 1, we use a three‐wave panel study to investigate whether loneliness and conspiracy mentality predict each other over time. In Study 2, we use a five‐wave panel study to investigate the relationship between loneliness and belief in specific conspiracy theories, namely Covid‐19 conspiracy beliefs. We expected that people who are, on average, lonelier are also more likely to have a stronger conspiracy mentality as well as stronger Covid‐19 conspiracy beliefs. Furthermore, we expected that people who feel lonelier than average at one time point are more likely to show greater conspiracy mentality as well as greater belief in Covid‐19 conspiracy theories at a subsequent time point, as well as vice versa (conspiracy beliefs predicting loneliness and loneliness predicting conspiracy beliefs). To investigate this question, we use a random‐intercept cross‐lagged panel model (RI‐CLPM, Hamaker et al., 2015). This model allows us to separate time‐invariant between‐person relationships from time‐variant within‐person processes, thereby identifying whether within‐person changes in one variable lead to subsequent within‐person changes in another variable.

## STUDY 1

### Method

The materials, analysis code, codebook, and Appendix S1 for Study 1 are available on our OSF project site (https://osf.io/5h4jf/). The preregistration of Study 1 was part of a larger project (for the current purpose, only research questions 1 and 2 are relevant) and can be found via this link: https://osf.io/akdw9. We report all data exclusions (if any) and all measures in the study. Data were analysed using R, version 4.3.0 (R Core Team, 2020), and the package *lavaan*, version 0.6‐15 (Rosseel, 2012).

#### Participants and design

The study used existing data from a larger longitudinal project investigating the psychological effects of the COVID‐19 pandemic in Germany across 16 waves. Here, we only focus on Waves 1, 3, and 5, as these waves include our variables of interest. Participants were recruited in diverse ways: via social media, press releases, and a link on the website of the institute for clinical psychology. Wave 1 took place from the 21st of December 2020 until the end of the study. Participation in Wave 1 was ongoing because whenever a new participant was recruited to the study, they started with the questionnaire from Wave 1 because it included all demographic questions. However, as preregistered, we only included participants who participated in Wave 1 before the 31st of March 2021. Table 1 displays an overview of the sample characteristics per wave. The sample was highly educated, with more than 70% of participants having at least a high school diploma. A total of 878 participants took part in all three waves, equipping us with the power of 0.80 to detect a medium‐sized (0.30) effect (see Appendix S1). All participants provided informed consent prior to participation in the study.

**TABLE 1:** Overview of measurement waves and corresponding timepoints of Study 1.

	Start	End	*N*	*M*_age_ (SD_age_)	Gender		
					Male	Female	Other
Wave 1	21.12.20	31.03.21	1604	39.55 (14.11)	355	1118	10
Wave 3	31.03.21	28.04.21	1143	41.2 (14.21)	248	852	8
Wave 5	23.06.21	21.07.21	917	42.58 (14.37)	186	702	8

#### Measures

##### Conspiracy mentality

We used the five‐item short version of the Conspiracy Mentality Scale (Imhoff, 2013) to measure conspiracy mentality. Participants answered on a 7‐point Likert scale, ranging from 1 (*completely disagree*) to 7 (*completely agree*), with higher values reflecting higher levels of conspiracy mentality. An example item is “*Most people do not recognise to what extent our lives are determined by conspiracies that are concocted in secret.”* The scale showed acceptable internal consistency (Cronbach's *ɑ* = .72).

##### Loneliness

To measure loneliness, we used the three‐item short version of the UCLA loneliness scale (Hughes et al., 2004). An example item is “How often do you feel that you are missing another person?” Participants answered on a 3‐point Likert scale, ranging from 1 (*rarely*) to 3 (*often*), with scores ranging from 3 to 9 and higher values representing greater loneliness. The scale showed good internal consistency (Cronbach's *α* = .76) and good convergent and divergent validity in past research (Hughes et al., 2004).

##### Demographics

Participants indicated their gender, age, education, and income.

#### Analytic strategy

To investigate reciprocal influences of variables over time, researchers typically use a cross‐lagged panel model (CLPM). The CLPM models how the deviation from the grand mean in one variable can lead to rank‐order change in another variable. However, recently, the CLPM was critiqued for not sufficiently differentiating between stable between‐person processes and time‐variant within‐person processes (Hamaker et al., 2015). Specifically, the CLPM assumes that all individuals are fluctuating around the same mean and that any deviation from this grand mean is meaningful. In many cases, however, it is plausible that individuals show at least some trait‐like individual differences, meaning that individuals are not fluctuating around a shared grand mean, but around an individual mean. Not distinguishing between stable between‐person processes and fluctuating within‐person processes could lead to biased parameter estimation, especially if the variables of interest are thought to show some stability (Usami et al., 2019). As both of our variables are, to some extent, more trait‐like (see Cacioppo et al., 2013; Imhoff et al., 2022), we expect the random intercept cross‐lagged panel model (RI‐CLPM) to be a more suitable model for our study.

**FIGURE 1:**
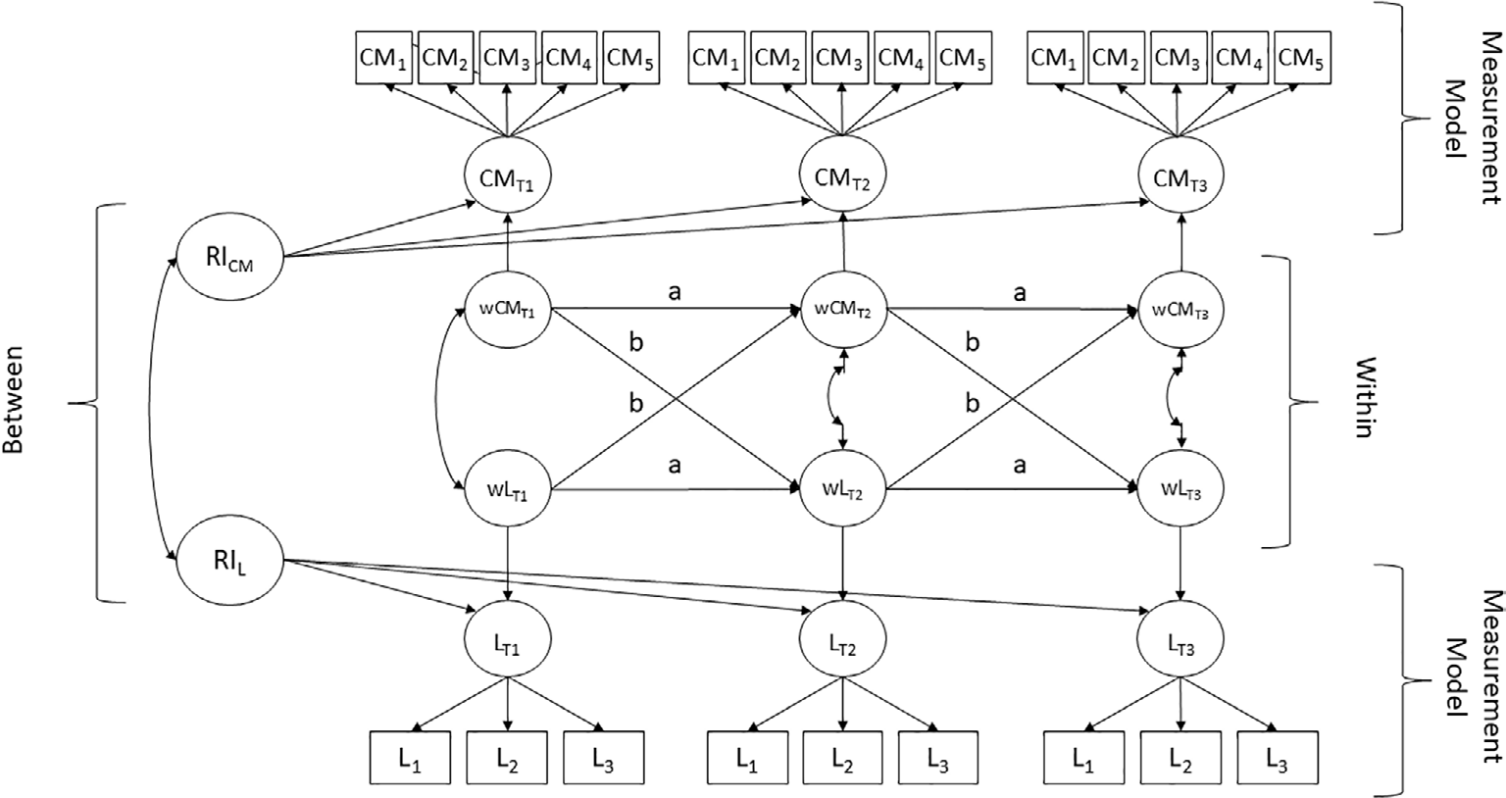
Random‐intercept cross‐lagged panel model for conspiracy mentality and loneliness. CM, conspiracy mentality; L, loneliness; RI, random intercept; w, within‐person component.

The RI‐CLPM expands the traditional CLPM by adding a latent random intercept to each indicator, thereby separating stable between‐person differences from situational within‐person effects (see Figure 1). This random intercept captures the stable between‐person variance, allowing individuals to vary around their individual mean instead of the grand mean. The correlations between the random intercepts tell us something about the relationship on the between‐person level. A positive correlation between the random intercepts, for example, would tell us whether people who are lonelier on average are also showing a higher conspiracy mentality.

The within‐person component, in comparison, models the within‐person change over time. The autoregressive effects of this model capture whether a deviation from an individual's mean can predict a deviation from this individual's mean at a subsequent timepoint (path a). For example, a positive autoregressive effect for loneliness would mean that if a person feels lonelier than they normally do, it is more likely that they also feel lonelier than they normally do at a subsequent time point. Similarly, the cross‐lagged effects capture whether a deviation from an individual's expected score in one variable predicts this individual's deviation from their expected score in another variable at a subsequent timepoint (path b). A positive cross‐lagged effect from loneliness to conspiracy mentality would imply that feeling lonelier than usual at one timepoint predicts scoring higher than usual in conspiracy mentality at a subsequent timepoint.

To analyse the data, we used the Full Information Maximum Likelihood (FIML) estimation to account for missing data because it has been shown to outperform other methods (Enders & Bandalos, 2001). To account for the non‐normal distribution of our variables, we used the robust Maximum Likelihood Estimator (MLR). As preregistered, we determined model fit using the Comparative Fit Index (CFI > 0.90), the Goodness of Fit Index (GFI > 0.90), the Root Mean Square Error of Approximation (RMSEA < 0.08), and the Standardised Root Mean Squared Residual (SRMR < 0.08; Hu & Bentler, 1999; McDonald & Ho, 2002).

### Results

#### Preliminary analyses

The means, standard deviations, and correlations for loneliness and conspiracy mentality of Wave 1 to 5 can be found in Table 2. We also tested for systematic dropout and longitudinal measurement invariance. For brevity, these analyses are detailed in the Appendix S1, and we only summarise the results here. We did observe some systematic dropout; most notably, participants high in conspiracy mentality were more likely to drop out after Wave 1. While this is hardly surprising in light of the association between conspiracy mentality and science scepticism, respectively distrust towards experts and institutions (Imhoff et al., 2018; Pummerer et al., 2021; Rutjens & Lee, 2020), it suggests our results should be interpreted with caution. For both the measure of loneliness and conspiracy mentality, we could assume at least (weak) longitudinal measurement invariance.

**TABLE 2:** Means, standard deviations, and zero‐order correlations for Study 1.

	*M*	SD	1.	2.	3.	4.	5.	6.
1. CM_1_	2.30	1.11	1	.7	.68	.08	.10	.10
2. CM_2_	2.29	1.05	.70	1	.74	.11	.10	.12
3. CM_3_	2.32	1.08	.68	.74	1	.10	.12	.14
4. Lonely_1_	6.35	1.78	.08	.11	.10	1	.71	.60
5. Lonely_2_	6.40	1.84	.10	.10	.12	.71	1	.68
6. Lonely_3_	5.72	1.88	.10	.12	.14	.60	.68	1

*Note*: *N* = 830 participants with no missing data of a total of *N* = 830 participants without missing data who took part in all waves, *r* > .05, *p* < .05; *r* > .08, *p* < .01; *r* > .11, *p* < .001.

##### Reciprocal influence of loneliness and conspiracy mentality

First, we fit the latent RI‐CLPM to our data. The model fitted the data well *χ* ^2^(220) = 446.94, *p* < .001, RMSEA = 0.03, CFI = 0.98, TLI = 0.98, SRMR = 0.04. Then, we tested whether a simple CLPM fits the data equally well by constraining the random intercepts to zero. The model fitted the data significantly worse (Δ*χ* ^2^(3) = 13.96, *p* < .01). Therefore, we assumed the RI‐CLPM to fit the data better. Next, we tested whether we could constrain the lagged parameters to be equal across time. The constrained model did not fit the data significantly worse (Δ*χ* ^2^(4) = 0.77, *p* = .94). Therefore, we could assume the lagged effects to be equal across time. The constrained model showed that the random intercepts of loneliness and conspiracy mentality were positively correlated, meaning that people who, on average, feel lonelier also have a higher conspiracy mentality (see Table 3 for results). Additionally, we observed a significant positive autoregressive effect for loneliness, indicating feeling lonelier than usual at one timepoint increases the probability to feel lonelier than average at a subsequent timepoint. However, neither the autoregressive effect for conspiracy mentality nor the cross‐lagged effects from conspiracy mentality to loneliness or vice versa were significant. The results remain unchanged once we use Bonferroni correction to account for multiple comparisons. As a robustness check, we investigated whether the results change if we only include participants who participated in wave 1 prior to 01.02.2021, thereby making the time intervals between the waves equal. Furthermore, we ran several additional models controlling for time‐invariant (age, gender, education) and time‐variant (social distancing strength, Covid‐19 fear) variables. The pattern of results did not change (see Appendix S1).

**TABLE 3:** Results of the RI‐CLPM between conspiracy mentality and loneliness.

	*B*	SE	*p*	*β*
**Autoregressions**				
Loneliness T1 → Loneliness T2	**0.49**	**0.11**	**<.001**	.**45**
Loneliness T2 → Loneliness T3	**0.49**	**0.11**	**<.001**	.**47**
CM T1 → CM T2	−0.12	0.35	.740	−.16
CM T2 → CM T3	−0.12	0.35	.740	−.07
**Cross‐Lagged Effects**				
Loneliness T1 → CM T2	−0.01	0.17	.975	−.01
Loneliness T2 → CM T3	−0.01	0.17	.975	−.01
CM T1 → Loneliness T2	0.09	0.14	.532	.08
CM T2 → Loneliness T3	0.09	0.14	.532	.05
**Within‐person correlations**				
Loneliness T1 ↔ CM T1	0.00	0.01	.975	.01
Loneliness T2 ↔ CM T2	−0.00	0.01	.896	−.03
Loneliness T3 ↔ CM T3	0.01	0.01	.338	.09
**Between‐person correlations**				
Loneliness ↔ CM	**0.05**	**0.01**	**<.001**	.**22**
**Model fit**	*X* ^2^(224) = 447.74, *p* < .001, RMSEA = 0.03, SRMR = 0.04, CFI = 0.98			

*Note*: Significant coefficients are written in bold.

Abbreviation: CM, conspiracy mentality.

### Discussion

This study supported prior evidence that people who feel, on average, lonelier are showing a stronger conspiracy mentality. However, we found no evidence that conspiracy mentality and loneliness predict each other over time. Additionally, we did not find evidence that showing higher than usual conspiracy mentality at one time point increases the probability of scoring higher than usual at a subsequent time point. One possible explanation for the null findings may be that we lacked statistical power to detect such an association. Given that our data included three measurements of almost 900 participants, it would suggest that should this null effect be due to low power, it is arguably very small. Another possibility is that we used a scale of conspiracy mentality as our indicator, which is rather stable and unlikely to change drastically over short periods of time. In comparison, belief in specific conspiracy theories is more time‐variant and malleable (Imhoff et al., 2022) and might thus be more suitable to test even subtle variations across time. To rule out that the null effects were due to a design choice of measure, we investigated the reciprocal relationship between specific conspiracy beliefs and loneliness in a second study with five time points.

## STUDY 2

### Method

Access to the dataset, full list of items, item wordings, and coding can be requested via the website of the Austrian Corona Panel Project (ACPP; https://viecer.univie.ac.at/coronapanel/austrian‐corona‐panel‐data/access‐request/). The analysis was preregistered, and the corresponding file can be accessed via this link: https://osf.io/da2w8. The analysis code is available on our OSF project website.

#### Participants and design

The data for this study stem from the ACPP, which investigates health, economic, and social aspects of the Corona crisis in Austria (Kittel et al., 2020a, 2020b). Data were collected in 34 waves, with the first wave starting on the 27th of March 2020 and wave 34 ending on the 27th of February 2023 (for exact time points, see Table 4). A total sample of *N* = 1541 participants were recruited via a pre‐existing online panel and were quota sampled based on age, gender, region, municipality size, and educational level to represent the Austrian population. To participate in the study, participants had to be at least 14 years old and reside in Austria. In the study at hand, we focus on waves 9, 15, 21, 23, and 30 because these include questions about conspiracy theories. A total of 649 respondents finished all waves included in this study, which provided us with a power of 0.98 to detect a medium‐sized effect (see Appendix S1). All participants provided informed consent before taking part in the study.

**TABLE 4:** Overview of measurement waves and corresponding timepoints of Study 2.

	Start	End	*N*	*M*_age_ (SD_age_)	Gender		
					Male	Female	Other
Wave 9	23.05.20	27.05.20	1502	46.34 (17.08)	738	758	6
Wave 15	11.09.20	18.09.20	1581	45.74 (16.91)	767	807	7
Wave 21	12.03.21	19.03.21	1573	46.91 (16.72)	780	786	7
Wave 23	21.05.21	28.05.21	1503	46.41 (16.69)	736	760	7
Wave 30	18.03.22	25.03.22	1507	45.29 (17.44)	759	741	7

#### Measures

##### Conspiracy beliefs

To measure belief in specific conspiracy theories, participants were asked how much they thought four different statements regarding conspiracy theories around the COVID‐19 pandemic were true. Specifically, participants were asked whether they thought that 5G transmitters were responsible for the coronavirus, that Bill Gates wants to force people to get the vaccine in order to make a profit, that the coronavirus is a bioweapon intentionally developed by humans, and that the coronavirus was accidentally released from a US military lab. Participants answered on a 5‐point Likert scale, ranging from 1 (*very certain that this is false*) to 5 (*very certain that this is true*), where higher values indicate a stronger belief in conspiracy theories. The internal consistency of the items was good (Cronbach's *ɑ* = .88).

##### Loneliness

Loneliness was measured using a single item. Participants were asked, “In the past week, how many times have you felt lonely?” The answer scale ranges from 1 (*never*) to 5 (*daily*).

##### Demographics

Participants indicated their gender, age, and education.

#### Analytic strategy

**FIGURE 2:**
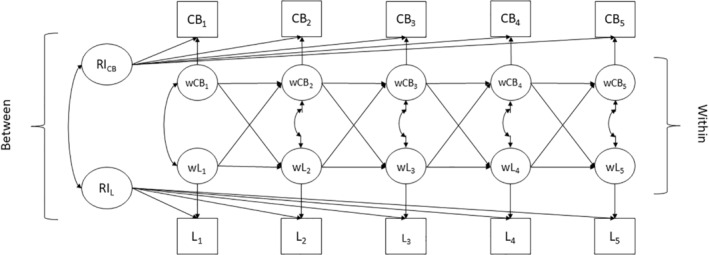
Random‐intercept cross‐lagged panel model between specific conspiracy beliefs and loneliness. CB, specific conspiracy beliefs; L, loneliness; RI, random intercept; w, within‐person component.

We conducted the same analyses reported in Study 1. The only difference was that we did not conduct a latent random‐intercept cross‐lagged panel model because loneliness was measured with one item only (see Figure 2).

### Results

#### Preliminary analyses

The means, standard deviations, and zero‐order correlations of loneliness and conspiracy belief can be found in Table 5. None of the variables used in this study predicted systematic dropout after Wave 9. Again, we could establish (weak) longitudinal measurement invariance for conspiracy beliefs (see Appendix S1).

**TABLE 5:** Means, standard deviations, and zero‐order correlation for Study 2.

	*M* (SD)	1.	2.	3.	4.	5.	6.	7.	8.	9.	10.
CB_1_	1.96 (0.86)	1	.72	.71	.62	.61	.05	.09	.07	.03	.04
CB_2_	1.93 (0.88)	.72	1	.70	.64	.61	.09	.13	.11	.06	.08
CB_3_	1.93 (0.88)	.71	.70	1	.71	.66	.04	.09	.08	.03	.08
CB_4_	1.91 (0.9)	.62	.64	.71	1	.64	.08	.11	.10	.04	.07
CB_5_	1.88 (0.89)	.61	.61	.66	.64	1	.1	.12	.12	.05	.15
Lonely_1_	1.53 (0.91)	.05	.09	.04	.08	.10	1	.60	.60	.61	.51
Lonely_2_	1.54 (0.96)	.09	.13	.09	.11	.12	.60	1	.57	.66	.58
Lonely_3_	1.67 (1.05)	.07	.11	.08	.10	.12	.60	.57	1	.67	.61
Lonely_4_	1.65 (1.06)	.03	.06	.03	.04	.05	.61	.66	.67	1	.61
Lonely_5_	1.68 (1.08)	.04	.08	.08	.07	.15	.51	.58	.61	.61	1

*Note*: *N* = 607 cases with no missing data, *r* > .08, *p* < .05; *r* > .10, *p* < .01; *r* > .13, *p* < .001.

#### Reciprocal influence of loneliness and conspiracy beliefs

The RI‐CLPM fitted the data well: *χ* ^2^(21) = 45.98, *p*  <.001, RMSEA = 0.04, CFI = 0.99, TLI = 0.99, SRMR = 0.02. Constraining the lagged parameters to be equal across waves significantly worsened the model fit (Δχ^2^(12) = 58.13, *p* < .001). Similarly, constraining the random intercepts to be zero, thereby making the model equivalent to a CLPM, significantly worsened the model fit (Δχ^2^(3) = 1028.8, *p* < .001). Therefore, we continued with the unconstrained RI‐CLPM model. As in Study 1, the random intercepts of loneliness and conspiracy beliefs correlated positively (see Table 6), indicating that people who, on average, feel lonelier also hold stronger conspiracy beliefs. Also in line with Study 1, loneliness showed a significant autoregressive effect over all five waves, with the autoregressive effects of the first waves being negative and the effects of the later waves being positive. For conspiracy beliefs, only the last two autoregressive effects are positive and significant. This means that for the later waves of the study, having stronger than usual conspiracy beliefs also predicts showing higher than usual conspiracy beliefs at a subsequent time point. None of the cross‐lagged effects from conspiracy beliefs to loneliness or vice versa became significant. As in Study 1, we ran robustness checks controlling for time‐invariant (age, gender, education) and time‐variant (face‐to‐face contact with friends or family, Covid‐19 threat) variables. The pattern of results did not change (see Appendix S1).

**TABLE 6:** Results of the RI‐CLPM between specific conspiracy beliefs and loneliness.

	*B*	SE	*p*	*β*
**Autoregressions**				
Loneliness T1 → Loneliness T2	**−0.21**	**0.07**	.**003**	**−.21**
Loneliness T2 → Loneliness T3	**−0.19**	**0.07**	.**008**	**−.15**
Loneliness T3 → Loneliness T4	**0.27**	**0.04**	**<.001**	.**27**
Loneliness T4 → Loneliness T5	**0.21**	**0.05**	**<.001**	.**19**
CB T1 → CB T2	0.08	0.05	.115	.08
CB T2 → CB T3	0.07	0.05	.136	.08
CB T3 → CB T4	**0.16**	**0.05**	.**001**	.**15**
CB T4 → CB T5	**0.15**	**0.05**	.**001**	.**15**
**Cross‐Lagged Effects**				
Loneliness T1 → CB T2	0.04	0.05	.441	.04
Loneliness T2 → CB T3	−0.04	0.05	.354	−.05
Loneliness T3 → CB T4	0.02	0.03	.626	.02
Loneliness T4 → CB T5	−0.04	0.04	.249	−.05
CB T1 → Loneliness T2	0.01	0.06	.917	.01
CB T2 → Loneliness T3	0.03	0.07	.708	.02
CB T3 → Loneliness T4	0.02	0.06	.716	.02
CB T4 → Loneliness T5	0.02	0.06	.689	.02
**Within‐person correlations**				
Loneliness T1 ↔ CB T1	0.00	0.01	.844	.01
Loneliness T2 ↔ CB T2	0.02	0.02	.225	.07
Loneliness T3 ↔ CB T3	0.00	0.02	.804	.01
Loneliness T4 ↔ CB T4	0.01	0.01	.425	.03
Loneliness T5 ↔ CB T5	**0.05**	**0.02**	.**001**	.**12**
**Between‐person correlations**				
Loneliness ↔ CB	**0.15**	**0.02**	**<.001**	.**25**
**Model fit**	*X* ^2^(21) = 45.98, *p* = .001, RMSEA = 0.02, SRMR = 0.02, CFI = 0.99			

*Note*: CB, belief in specific conspiracy theories. Significant coefficients are written in bold.

Exploratorily, we ran another RI‐CLPM including three instead of five measurement occasions, with each measurement point being roughly 10–12 months apart from each other. This model allowed us to investigate whether time intervals were chosen too close together to observe change. Again, we found a positive between‐person correlation between loneliness and specific conspiracy beliefs (*r* = .29, *p* = <.001), and we did not find evidence for a cross‐lagged effect between specific conspiracy beliefs and loneliness. However, we did find an autoregressive effect for both specific conspiracy beliefs (*B* = 0.26, *p* < .001) and loneliness (*B* = 0.45, *p* < .001; see Appendix S1).

### Discussion

The aim of Study 2 was to test whether the results of Study 1 were partly due to conspiracy mentality being resistant to change. Therefore, Study 2 looked at the relationship between loneliness and specific conspiracy beliefs. Study 2 replicated the main results of Study 1: Again, we found that people who, on average, feel lonelier are also showing stronger beliefs in specific conspiracy theories, and we did not find evidence for the idea that conspiracy beliefs and loneliness predict each other over time. However, we did find more mixed patterns for the autoregressive effects for loneliness and conspiracy beliefs: The autoregressive effect for loneliness was negative for the first two lags and became positive for the last two lags. The autoregressive effect for conspiracy beliefs was non‐significant for the first two lags and became positive for the last two lags. The mixed results for the autoregressive effects could be the results of the generally dynamic process of the COVID‐19 pandemic unfolding during the measurement waves, in which lockdown measures differed per country and measurement occasion. The difference in the autoregressive effects in Study 1 and Study 2 could have been caused by the different time intervals, with Study 1 taking place from December 2020 until July 2021 and Study 2 taking place from May 2020 until March 2022. Generally, these results join previous findings in providing no support for the notion that conspiracy beliefs and loneliness form a vicious cycle.

## GENERAL DISCUSSION

In two longitudinal studies, we set out to investigate the relationship between two constructs with possibly grave consequences: conspiracy beliefs and loneliness. Specifically, we investigated whether loneliness and conspiracy beliefs predict each other over time, thereby potentially forming a vicious cycle. Both studies showed that the two variables show a small to medium positive relationship on the between‐person level: People who feel lonelier on average are also showing a greater conspiracy mentality as well as greater belief in specific conspiracy theories. This is in line with previous studies showing a positive cross‐sectional relationship between the two variables under investigation (e.g., Neu et al., 2023). However, neither study supported a relationship between the two constructs on the within‐person level, meaning that people who feel lonelier than they usually do are not more likely to adopt conspiracy beliefs at a subsequent timepoint or vice versa. Therefore, we did not find evidence for a vicious cycle. This finding is in line with previous failures to show that inducing feelings of loneliness influences conspiracy beliefs (Jolley et al., 2023).

The fact that we found a cross‐sectional but not a longitudinal relationship between loneliness and conspiracy beliefs can be explained in various ways. It is possible that the between‐person relationship we find between conspiracy beliefs and loneliness is due to an unobserved third variable that causes both loneliness and conspiracy beliefs to be correlated. For example, two variables that are known to correlate with both conspiracy beliefs and loneliness are subclinical paranoia (Alsuhibani et al., 2022; Imhoff & Lamberty, 2018) and socio‐economic status (Adam‐Troian et al., 2022; de Jong Gierveld et al., 2015; van Prooijen, 2016 ). Controlling these variables while investigating the relationship between conspiracy beliefs and loneliness could diminish the relationship observed in our study, as has already been observed in other cross‐sectional studies (Alsuhibani et al., 2022).

It is also possible that the unique situation during the Covid‐19 pandemic caused dynamic shifts in within‐person changes, thereby hindering us from observing a cross‐lagged relationship between our conspiracy variables and loneliness (Entringer & Gosling, 2022; Kung et al., 2023; van Mulukom et al., 2022). For example, our first two measurement waves of Study 1 took place during a time of rising 7‐day incidences of Covid‐19 infections and increased preventive measurement, while the last measurement point took place during a time of decreasing case numbers and easing of measures (DPA, 2023, February 2). These partly individually (in case of mandatory quarantine), regionally, and temporally differing preventive measures could affect loneliness (Ernst et al., 2022), which again could suppress the relationship between our measures of conspiracy beliefs and loneliness. Even though our robustness analyses indicated no such suppressing effects when controlling for social distancing strength and contact with friends and family, replication studies outside of the Covid‐19 pandemic are necessary to thoroughly exclude this possibility.

Another explanation for our pattern of results could be that it is not the transient, adaptive feeling of loneliness that most of us experience throughout our lives, but the persistent and maladaptive loneliness that is related to conspiracy beliefs. Generally, loneliness is thought to be an adaptive feeling that temporarily causes withdrawal from social interaction and increased social threat perception, followed by a motivation to reaffiliate (Cacioppo et al., 2015). However, for a small proportion of people, this reaffiliation does not happen, and instead, people feel prolonged loneliness (Dulmen & Goossens, 2013; Qualter et al., 2015). It follows then that the negative cognitive biases associated with loneliness, including the hypervigilance for social cues and social threat perception, are also prolongedly activated. This long‐term uncomfortable state could increase the need to reappraise one's painful feeling as not only caused by chance or oneself but also by the sinister intentions of others. After all, if loneliness were caused only by oneself, one would be able to decrease it. In line with this reasoning, prolonged loneliness is associated with lower trust in others (Qualter et al., 2013) and increased attribution of negative intentions to others (Okruszek et al., 2021; Qualter et al., 2012). Both trust in others (Meuer & Imhoff, 2021) and the ascription of negative intention to others (Frenken & Imhoff, 2022) have also been identified as robust correlates of conspiracy mentality.

### Limitation and future research

While our study shows considerable advantages, such as its pre‐registered analysis plans, the longitudinal nature of the data, the establishment of measurement invariance across measurement waves, and large sample sizes, it also comes with limitations. First, our measure for loneliness in Study 2 was not ideal. Future research should use validated measures of loneliness that ideally also capture its different facets, allowing for the investigation of the relationship between conspiracy beliefs and isolated, relational, as well as collective loneliness (see Hawkley et al., 2005).

Second, it is possible that the true within‐person cross‐lagged effect sizes between our conspiracy variables and loneliness are too small to be detected in our study. A recent study conducted in New Zealand with more than 55,000 participants was able to detect small cross‐lagged effects between psychological needs and conspiracy beliefs. Researchers should keep these effect sizes in mind when designing future studies (Albath et al., 2024).

Third, in longitudinal research, uncovering relationships relies on choosing appropriate measurement intervals. If the intervals are chosen too close to each other, change might not have happened yet, whereas if chosen too far apart, change might not be appropriately modelled (Bollen, 1989). In Study 2, we ran two different RI‐CLPM models, one with three measurement points roughly 10 months apart and one with five measurement points closer to each other. Only for the three‐wave RI‐CLPM did we find an autoregressive effect for specific conspiracy beliefs. This could be an indication that change in specific conspiracy beliefs needs time intervals of 10 months or more to be observed. Given that conspiracy mentality is known to be even more stable than specific conspiracy belief (Imhoff et al., 2022), our time intervals might have been too short to witness change in conspiracy mentality. However, other research using smaller time intervals does find cross‐lagged relationships between specific conspiracy beliefs/conspiracy mentality and their variables of interest (see Liekefett, 2021). Future research should investigate which time intervals are appropriate to capture change for both specific conspiracy beliefs and conspiracy mentality (Liekefett et al., 2021).

And lastly, our study uses samples from two very similar and WEIRD countries (Henrich et al., 2010), namely Austria and Germany. Cultural differences have so far received only little attention in research on conspiracy beliefs (Imhoff, 2022; but see Adam‐Troian et al., 2021, Alper & Imhoff, 2023), but many associations (or lack thereof) might not hold up in other contexts. As an example, the typical negative correlation between conspiracy beliefs and institutional trust reverses in the Russian context, where many conspiracy theories are spread by the state (Bogatyreva, 2024). More research in other contexts is thus needed to deeply understand the relationship between loneliness and conspiracy beliefs. For example, it is possible that in countries where conspiracy beliefs are spread by the government, where conspiracy beliefs are more mainstream, conspiracy beliefs could decrease loneliness because it makes people feel attitudinally closer to those around them.

### Conclusion

Thus far, the evidence for the relationship between loneliness and conspiracy beliefs was mixed, and the directionality of the relationship between the two was unclear. In two longitudinal studies, we found a cross‐sectional relationship between conspiracy beliefs and loneliness but no evidence for a relationship over time. This indicates that in the general population, lonely people are more likely to believe in conspiracy theories; however, we do not find evidence for a vicious cycle. This finding adds important insights to the literature on need deprivation and conspiracy beliefs.

## AUTHOR CONTRIBUTIONS

**Tisa Bertlich:** Conceptualization; formal analysis; writing – original draft; data curation. **Anne‐Kathrin Bräscher:** Conceptualization; data curation; investigation; project administration; writing – review and editing. **Sylvan Germer:** Investigation; data curation; writing – review and editing; project administration. **Michael Witthöft:** Conceptualization; methodology; supervision; writing – review and editing. **Roland Imhoff:** Conceptualization; supervision; writing – review and editing.

## CONFLICT OF INTEREST STATEMENT

The authors declare no conflicts of interest.

## Supporting information


Appendix S1.

## Data Availability

The data that support the findings of Study 1 are openly available at the OSF at https://osf.io/5h4jf/?view_only=d070b506cb7e43949a33972a2b79cb33. The data that support the findings of Study 2 are available from the Austrian Corona Panel Project. Restrictions apply to the availability of these data, which were used under license for this study. Data are available from https://viecer.univie.ac.at/coronapanel/austrian‐corona‐panel‐data/method‐report/ with the permission of the Austrian Corona Panel Project.
