# Supplementary material for: Owners of a conspiratorial heart? Investigating the longitudinal relationship between loneliness and conspiracy beliefs
Source: Br J Soc Psychol. 2025 Feb 20;64(2):e12865. doi: 10.1111/bjso.12865 (PMC11840883; doi:10.1111/bjso.12865)
Supplement: Supplementary file 1 — Appendix S1. [file BJSO-64-0-s001.pdf]

Supplementary Online Material

Study 1

Sample

Participants were recruited in diverse ways: via social media, press releases, a link on the website of the institute for clinical psychology. Wave 1 took place from 21st of December 2020 until the end of the study. Participation in Wave 1 was ongoing because whenever a new participant was recruited to the study, they started with the questionnaire from Wave 1 because it included all demographic questions. However, as preregistered, we only included participants who participated in Wave 1 before the 31st of March 2021. To investigate the power of our study, we ran Monte-Carlo simulations as proposed by Mulder (2022). We ran the simulation for our sample size of $N = 878$, small ($\beta_1 = .30$) and medium-sized autoregressive and cross-lagged effect ($\beta_2 = .30$), a within-person correlation of .1, an intraclass correlation coefficients of .7, a random intercept of .2, three measurement occasions, and 1000 repetitions. The corresponding analysis script can be found on the OSF project website. The simulation indicated that our power was sufficient (.80 –.94) for medium effect sizes and low (.36 - 47) for small effect sizes.

Measures

We measured social distancing strength by asking participants "How much are you currently reducing your social contacts?" Answers were given on a 5-point Likert scale, ranging from *1* (not at all) to *5* (very much). To tap into fear of Covid-19, we asked participants the following four questions "How afraid are you of dying from COVID-19?", "How great is your fear of relatives or close friends die from COVID-19?", and "How strong is your fear of becoming infected with the coronavirus?". Participants answered on a 10-point Likert scale, ranging from *1* (not strong at all) to *10* (very strong).

Dropout Analysis

We also checked whether dropout from our study was systematic. To do so, we first ran a logistic regression predicting dropout after Wave 1 using loneliness, conspiracy mentality, and our demographic variables. Second, we ran a logistic regression predicting dropout after Wave 3 using the

same variables as above. We found that younger participants (B = -0.03, SE = .007, p < .01) and participants higher in conspiracy mentality (B = 0.13, SE = .06, p = .04) were more likely to drop out after the Wave 1, and younger (B = -0.03, SE = .007, p < .01) and male (B = 0.39, SE = .17, p = .03) participants were more likely to drop out after Wave 3. The finding that people higher in conspiracy mentality are more likely to drop out of our study is not surprising: Previous studies show that conspiracy mentality is associated with science skepticism and distrust towards experts and institutions (Pummerer et al., 2021; Rutjens & Van der Lee, 2020), which could reduce participants motivation to take part in the study. Nevertheless, due to the dropout, our results should be interpreted with caution.

Longitudinal Measurement Invariance

To test whether our items are measuring the same construct over time, we investigated the longitudinal measurement invariance of our variables (Mackinnon et al., 2022). This is done in multiple steps: First, we tested whether the same factor model fitted the data at all time points (configural model). Next, we tested whether we could constrain the factor loadings to be equal across timepoints (metric or weak model). To compare associations over time, it is assumed that weak invariance is enough (van de Schoot et al., 2012). We compared the different models to each other using a chi square difference test. However, with large sample sizes, the chi square difference test can become significant even with minor differences (Cheung & Rensvold, 2002). Therefore, additionally, we assume a model to fit if the change in CFI, RMSEA and SRMR is not greater than a set value (ΔCFI <= -.01, ΔRMSEA <= .15, and ΔSRMR <= .03, see Chen, 2007). If the difference between the two models is significant and the difference for CFI, RMSEA, and SRMR is beyond the threshold, the less parsimonious (less constrained) model should be chosen. The model results for the longitudinal measurement invariance models are depicted In Table A1. We could assume weak longitudinal measurement invariance to hold.

Robustness Analysis

As a robustness analysis, we investigated whether the results of the constrained RI-CLPM changed once we only included participants who joined the study before 01.02.2021, thereby making all three waves equally long and equally spaced apart. Generally, the pattern of results stayed the

same: The model fitted the data well $\chi^2(224) = 394.78$, $p < .001$, RMSEA = .03, CFI = .98, TLI = .98, SRMR = .04. We observed an autoregressive effect for loneliness (*B* = .44, *SE* = .12, *p* < .001, 95% CI = [.20, .67]), meaning that people who feel lonelier than they usually do are also more likely to feel lonely than they usually do at a subsequent timepoint. We did not find evidence for an autoregressive effect for conspiracy mentality (*B* = -.18, *SE* = .26, *p* = .50, 95% CI = [-.69, .34]), not for a cross-lagged effect form loneliness to conspiracy mentality (*B* = -.07, *SE* = .20, *p* = .74., 95% CI = [-.46 .33]) or vice versa (*B* = .03, *SE* = .16, *p* = .85, 95% CI = [-.28, .34]).

Table A1

Model fit and Model Comparison for the Measurement Invariance Models

	X^2	df	*p*	CFI	GFI	RMSEA	SRMR
**Conspiracy Mentality**							
Configural	204.411	66	< .001	.968	.967	.051	.043
Weak	213.775	74	< .001	.968	.965	.048	.047
Δ	9.363	8	.313				
**Loneliness**							
Configural	43.915	15	< .001	.993	.989	.047	.025
Weak	48.432	19	< .001	.993	.988	.042	.030
Δ	4.518	4	.34				

Additionally, we ran three robustness RI-CLPM. In the first model, we investigated the reciprocal relationship between loneliness and conspiracy mentality while controlling for the time-invariant covariates age, gender and education. We included the covariates by allowing them to correlate with the factor model of loneliness and conspiracy mentality at each time point (see Figure A1). In the second model, we ran a more complex RI-CLPM investigating the reciprocal relationship between the three variables conspiracy mentality, loneliness and adherence to social distancing measures while controlling for age, gender, and education. In the last RI-CLPM, we investigated the

reciprocal relationship between conspiracy mentality, loneliness, and fear of a Covid-19 infection, still controlling for gender, age and education. All models fitted the data well, and the overall pattern of results stayed similar to the original, preregistered model (see Tables A2, A3, A4). In none of the models, there was a cross-lagged effect between loneliness and conspiracy mentality or vice versa.

Figure A1

Latent RI-CLPM Between Loneliness and Conspiracy Mentality Including Covariates

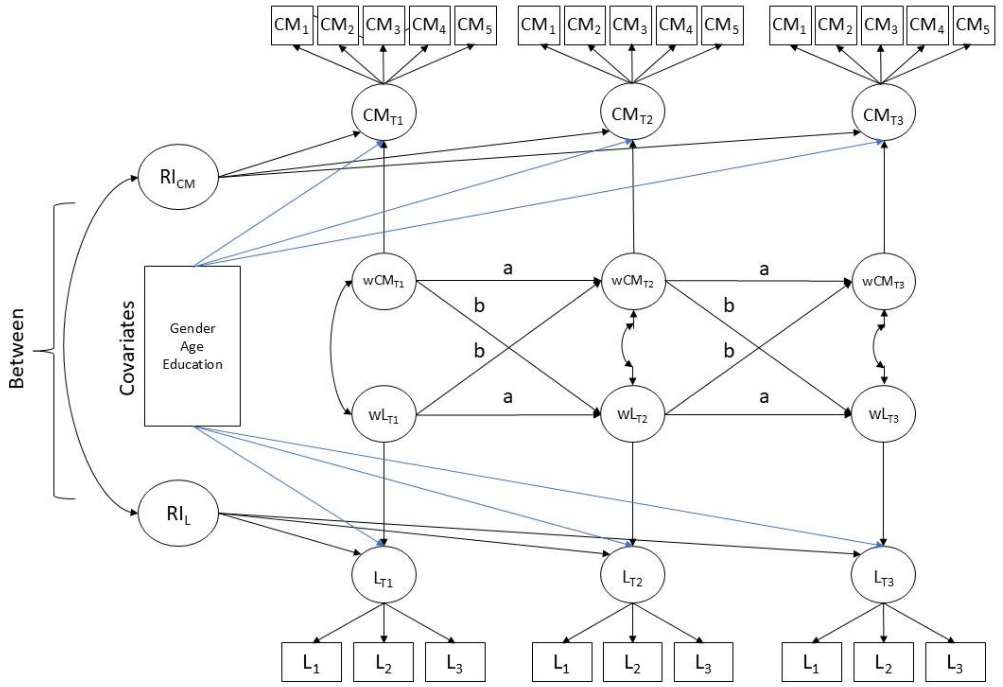

Table A2

Results of the RI-CLPM Between Loneliness and Conspiracy Mentality Controlling for Gender, Age, and Education

	B	SE	*p*	β
**Autoregressions**				
Loneliness T1 → Loneliness T2	**0.424**	**.123**	**.001**	**.390**
Loneliness T2 → Loneliness T3	**0.424**	**.123**	**.001**	**.400**
CM T1 → CM T2	-0.079	.448	.860	-.120
CM T2 → CM T3	-0.079	.448	.860	-.044
**Cross-Lagged Effects**				
Loneliness T1 → CM T2	-0.000	.210	.999	-.000
Loneliness T2 → CM T3	-0.000	.210	.999	-.000
CM T1 → Loneliness T2	0.059	.158	.708	.059
CM T2 → Loneliness T3	0.059	.158	.708	.037
**Within-person correlations**				
Loneliness T1 ↔ CM T1	0.000	.011	.988	.002
Loneliness T2 ↔ CM T2	-0.002	.015	.884	-.048
Loneliness T3 ↔ CM T3	0.008	.008	.354	.090
**Between-person correlations**				
Loneliness ↔ CM	**0.039**	**0.015**	**0.011**	**0.161**
**Model fit**				
	$X^2(278) = 578.13$, $p < .001$, RMSEA = .03, SRMR = .04, CFI = .97			

Table A3

Results of the Constrained Three-wave RI-CLPM Between Loneliness, Conspiracy Mentality, and Social Distancing Adherence Controlling for Gender, Age, and Education

	B	SE	*p*	β
**Autoregressions**				
Loneliness T1 → Loneliness T2	**0.388**	**.123**	**.002**	**.358**
Loneliness T2 → Loneliness T3	**0.388**	**.123**	**.002**	**.365**
CM T1 → CM T2	-0.103	.392	.793	-.168
CM T2 → CM T3	-0.103	.392	.793	-.053
Social Distancing T1 → Social Distancing T2	**0.359**	**.086**	**<.001**	**.298**
Social Distancing T2 → Social Distancing T3	**0.359**	**.086**	**<.001**	**.236**
**Cross-Lagged Effects**				
Loneliness T1 → CM T2	0.007	.213	.975	.010
Loneliness T2 → CM T3	0.007	.213	.975	.006
Loneliness T1 → Social Distancing T2	-0.046	.193	.813	-.017
Loneliness T2 → Social Distancing T3	-0.046	.193	.813	-.012
CM T1 → Loneliness T2	0.031	.168	.854	.032
CM T2 → Loneliness T3	0.031	.168	.854	.018
CM T1 → Social Distancing T2	-0.296	.362	.414	-.125
CM T2 → Social Distancing T3	-0.296	.362	.414	-.050
Social Distancing T1 → Loneliness T2	0.040	.029	.170	.081
Social Distancing T2 → Loneliness T3	0.040	.029	.170	.091
Social Distancing T1 → CM T2	-0.072	.072	.319	-.230
Social Distancing T2 → CM T3	-0.072	.072	.319	-.142
**Within-person correlations**				
CM T1 ←→ Loneliness T1	-0.001	.012	.933	-.014
CM T1 ←→ Social Distancing T1	-0.024	.020	.232	-.159
Loneliness T1 ←→ Social Distancing T1	0.012	.016	.431	.090
CM T2 ←→ Loneliness T2	-0.004	.015	.775	-.105
CM T2 ←→ Social Distancing T2	-0.019	.033	.559	-.191
Loneliness T2 ←→ Social Distancing T2	**0.028**	**.011**	**.012**	**.179**
CM T3 ←→ Loneliness T3	0.007	.008	.390	.080
CM T3 ←→ Social Distancing T3	-0.001	.022	.965	-.003
Loneliness T3 ←→ Social Distancing T3	**0.121**	**.014**	**<.001**	**.470**
**Between-person correlations**				
Loneliness ←→ CM	**0.042**	**.016**	**.007**	**.169**
CM ←→ Social Distancing	**-0.141**	**.035**	**<.001**	**-.337**
Social Distancing ←→ Loneliness	0.031	.016	.053	.159
**Model fit**				
	$X^2(357) = 739.46, p < .001$, RMSEA = .03, SRMR = .04, CFI = .97			

Table A4

Results of the Constrained Three-wave RI-CLPM Between Loneliness, Conspiracy Mentality, and COVID-19 Fear Controlling for Gender, Age, and Education

	B	SE	*p*	β
**Autoregressions**				
Loneliness T1 → Loneliness T2	**0.434**	**.126**	**.001**	**.397**
Loneliness T2 → Loneliness T3	**0.434**	**.126**	**.001**	**.412**
CM T1 → CM T2	0.742	.498	.136	.716
CM T2 → CM T3	0.742	.498	.136	.691
Cov-19 Fear T1 → Cov-19 Fear T2	**0.582**	**.099**	**<.001**	**.550**
Cov-19 Fear T2 → Cov-19 Fear T3	**0.582**	**.099**	**<.001**	**.565**
**Cross-Lagged Effects**				
Loneliness T1 → CM T2	-0.026	.124	.834	-.015
Loneliness T2 → CM T3	-0.026	.124	.834	-.015
Loneliness T1 → Cov-19 Fear T2	-0.136	.505	.788	-.018
Loneliness T2 → Cov-19 Fear T3	-0.136	.505	.788	-.019
CM T1 → Loneliness T2	-0.012	.034	.710	-.034
CM T2 → Loneliness T3	-0.012	.034	.710	-.019
CM T1 → Cov-19 Fear T2	0.834	.853	.329	.189
CM T2 → Cov-19 Fear T3	0.834	.853	.329	.190
Cov-19 Fear T1 → Loneliness T2	0.014	.015	.362	.089
Cov-19 Fear T2 → Loneliness T3	0.014	.015	.362	.090
Cov-19 Fear T1 → CM T2	0.017	.029	.551	.069
Cov-19 Fear T2 → CM T3	0.017	.029	.551	.069
**Within-person correlations**				
CM T1 ←→ Loneliness T1	-0.015	.025	.543	-.139
CM T1 ←→ Cov-19 Fear T1	0.214	.446	.631	.273
Loneliness T1 ←→ Cov-19 Fear T1	0.008	.092	.933	.017
CM T2 ←→ Loneliness T2	-0.004	.008	.660	-.047
CM T2 ←→ Cov-19 Fear T2	-0.018	.043	.671	-.041
Loneliness T2 ←→ Cov-19 Fear T2	0.042	.027	.120	.112
CM T3 ←→ Loneliness T3	0.005	.008	.526	.057
CM T3 ←→ Cov-19 Fear T3	-0.011	.055	.842	-.021
Loneliness T3 ←→ Cov-19 Fear T3	0.039	.027	.149	.098
**Between-person correlations**				
Loneliness ←→ CM	**0.052**	**.026**	**.049**	**.250**
Cov-19 Fear ←→ CM	-0.583	.461	.206	-.461
Cov-19 Fear ←→ Loneliness	0.098	.090	.275	.144
**Model fit**	$X^2(645) = 1566.08, p < .001$, RMSEA = .03, SRMR = .06, CFI = .96			

Study 2

Power Analysis

We assessed the achieved power of our study by running simulations described in the SOM of Study 1. We ran simulation with our sample size of $N = 649$ assuming small and medium autoregressive and cross-lagged effect sizes ($\beta_1 = .15$, $\beta_2 = .30$), a within-person correlation of .1, an intraclass correlation coefficients of .7, a random intercept of .2, five measurement occasions, and 1000 repetitions. Again, the analysis script is stored on OSF. The simulation indicated that our power was excellent (> .98) to detect a medium-sized effect and good (.64 - .75) to detect a small-sized effect.

Measures

The following items were used to measure belief in specific conspiracy theories: "*The new 5G transmitter masts are responsible for the spread of the coronavirus*", "*Bill Gates wants to vaccinate humanity by force in order to earn a lot of money.*", "*The coronavirus is a bioweapon that was deliberately developed to harm humans.*" and "*The coronavirus was accidentally released during a secret US military experiment*."

Contact with other people was measures using the item "Have you left your home last week for the following reasons? Meeting friends or family". Participants could answer on a 5-point Likert scale ranging from 1 (never) to 5 (daily). Perceived health risk was measured by asking participants how great they estimate the health risk posed by the coronavirus to them personally. Participants could answer on a 5-point Likert scale ranging from *1* (very large) to *5* (very small).

Longitudinal Measurement Invariance

As in Study 1, we investigated the longitudinal measurement invariance of our variables. This time, we only focused on conspiracy beliefs because loneliness consisted of one item only, making it impossible to investigate measurement invariance. The configural model for conspiracy beliefs fitted the data well ($\chi2(120) = 317.352$, $p < .001$, RMSEA = .05, CFI = .98, GFI = .95, SRMR = .04.). When constraining the factor loadings to be equal across time, the model fit is still good, and the models do

not differ significantly from each other (weak model: $\chi 2(132) = 325.90$, $p < .001$, RMSEA = .05, CFI = .98, GFI = .95, SRMR = .04; $\Delta\chi 2(12) = 8.55$, $p = .74$).

Robustness Analysis

Analogue to Study 1, we ran additional RI-CLPM as a robustness check. First, we ran a model similar to our preregistered model with the addition that we included the time-invariant covariates age, gender, and education. We included the covariates by allowing them to correlate with every observation of loneliness and belief in specific conspiracy theories at each time point (see Figure A2). The model fitted the data well, and the overall pattern of results stayed the same (see Table A5). As a second robustness analysis, we calculated two RI-CLPM with conspiracy beliefs and loneliness including only three measurement points, roughly 10 to 12 months apart (Model 1: wave 9, 23 and 30; Model 2: wave 9, 21, 23 of the Vienna Corona Panel Project). We tested whether constraining the autoregressive and cross-legged effect would worsen the model fit. We did not find evidence for a worse model fit (Model 1: X2(4) = 1.06, p = 901, Model 2: X2(4) = 2.46, p = .652). The results of the constrained models are displayed in Table A6 and Table A7. Interestingly, in three-wave models, the autoregressive effects of loneliness and belief in specific conspiracy theories become unidirectional and significant.

Figure A2

RI-CLPM Between Loneliness and Belief in Specific Conspiracy Theories Including Covariates

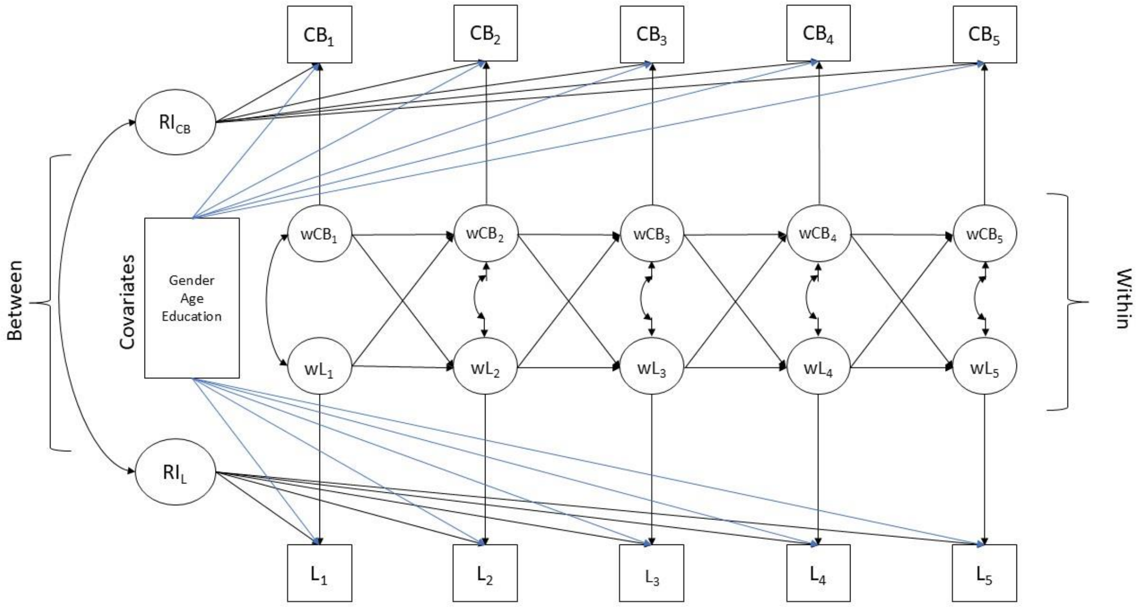
CB1
CB2
CB3
CB4
CB5
RICB
Between
Covariates
Gender
Age
Education
wCB1
wCB2
wCB3
wCB4
wCB5
wL1
wL2
wL3
wL4
wL5
Within
RIL
L1
L2
L3
L4
L5

As a third robustness analysis, we calculated a RI-CLPM with conspiracy beliefs, loneliness, and contact with other people including three measurement points (wave 9, 23 and 30 of the Vienna Corona Panel Project). The item to measure contact with others read “Have you left your home last week for the following reasons: Meeting friends and family?” Answers were given on a Likert-type scale, ranging from 1 (*never*) to 5 (*daily*)). We tested whether constraining the autoregressive and cross-legged effect would worsen the model fit. We did not find evidence for a worse model fit ($X^2(9) = 6.52$, p = .687). Therefore, we proceeded with the constrained model. Again, the general relationship between conspiracy beliefs and loneliness stayed the same (see Table A8). And lastly, we calculated a RI-CLPM with conspiracy beliefs, loneliness, and the perceived health risk the Covid-19 virus poses including three measurement points (wave 9, 23 and 30 of the Vienna Corona Panel Project). The item to measure perceived health risk read “How great do you estimate the health risk posed by the coronavirus to you personally and to the Austrian population?” Answers were given on a Likert-type scale, ranging from 1 (*very large*) to 5 (*very small*)). Again, the general relationship between conspiracy beliefs and loneliness stayed the same (see Table A9).

Table A5

Results of the RI-CLPM Between Specific Conspiracy Beliefs and Loneliness Controlling for Gender, Age, and Education

	B	SE	*p*	β
**Autoregressions**				
Loneliness T1 → Loneliness T2	**-0.204**	**0.068**	**.003**	**-0.204**
Loneliness T2 → Loneliness T3	**-0.196**	**0.071**	**.006**	**-0.156**
Loneliness T3 → Loneliness T4	**0.276**	**0.040**	**< .001**	**0.276**
Loneliness T4 → Loneliness T5	**0.212**	**0.050**	**< .001**	**0.194**
CB T1 → CB T2	0.077	0.051	.130	0.077
CB T2 → CB T3	0.081	0.050	.105	0.082
CB T3 → CB T4	**0.168**	**0.050**	**.001**	**0.151**
CB T4 → CB T5	**0.152**	**0.044**	**.001**	**0.153**
**Cross-Lagged Effects**				
Loneliness T1 → CB T2	0.044	0.051	.390	0.046
Loneliness T2 → CB T3	-0.022	0.047	.639	-0.024
Loneliness T3 → CB T4	0.019	0.033	.575	0.023
Loneliness T4 → CB T5	-0.048	0.036	.186	-0.059
CB T1 → Loneliness T2	0.003	0.064	.960	0.003
CB T2 → Loneliness T3	0.035	0.071	.621	0.026
CB T3 → Loneliness T4	0.028	0.057	.629	0.020
CB T4 → Loneliness T5	0.014	0.058	.809	0.011
**Within-person correlations**				
Loneliness T1 ←→ CB T1	-0.002	0.013	.863	-0.008
Loneliness T2 ←→ CB T2	0.023	0.015	.125	0.089
Loneliness T3 ←→ CB T3	0.009	0.015	.542	0.028
Loneliness T4 ←→ CB T4	0.008	0.013	.506	0.024
Loneliness T5 ←→ CB T5	**0.042**	**0.015**	**.005**	**0.107**
**Between-person correlations**				
Loneliness ←→ CB	**0.111**	**0.015**	**< .001**	**0.206**
**Model fit**				
	$X^2(21) = 44.86$, $p = .002$, RMSEA = .02, SRMR = .02, CFI = .99			

Table A6

Results of the Constrained Three-wave RI-CLPM Between Specific Conspiracy Beliefs and Loneliness Controlling for Gender, Age, and Education (including wave 9, 23 and 30)

	B	SE	*p*	β
**Autoregressions**				
Loneliness T1 → Loneliness T2	**0.448**	**0.051**	**< .001**	**.329**
Loneliness T2 → Loneliness T3	**0.448**	**0.051**	**< .001**	**.442**
CB T1 → CB T2	**0.258**	**0.060**	**< .001**	**.228**
CB T2 → CB T3	**0.258**	**0.060**	**< .001**	**.271**
**Cross-Lagged Effects**				
Loneliness T1 → CB T2	0.013	0.036	.724	.012
Loneliness T2 → CB T3	0.013	0.036	.724	.018
CB T1 → Loneliness T2	0.044	0.057	.437	.029
CB T2 → Loneliness T3	0.044	0.057	.437	.032
**Within-person correlations**				
Loneliness T1 ←→ CB T1	-0.018	0.022	.408	-.057
Loneliness T2 ←→ CB T2	**0.046**	**0.021**	**.033**	**.100**
Loneliness T3 ←→ CB T3	**0.047**	**0.017**	**.006**	**.114**
**Between-person correlations**				
Loneliness ←→ CB	**0.144**	**0.026**	**<.001**	**.285**
**Model fit**				
	$X^2(5) = 1.58$, $p = .904$, RMSEA < .01, SRMR = .01, CFI = .99			

Table A7

Results of the Constrained Three-wave RI-CLPM Between Specific Conspiracy Beliefs and Loneliness Controlling for Gender, Age, and Education (including wave 9, 21 and 30)

	B	SE	*p*	β
**Autoregressions**				
Loneliness T1 → Loneliness T2	**0.294**	**0.060**	**<.001**	**0.224**
Loneliness T2 → Loneliness T3	**0.294**	**0.060**	**<.001**	**0.282**
CB T1 → CB T2	**0.256**	**0.068**	**<.001**	**0.243**
CB T2 → CB T3	**0.256**	**0.068**	**<.001**	**0.249**
**Cross-Lagged Effects**				
Loneliness T1 → CB T2	-0.011	0.040	0.780	-0.011
Loneliness T2 → CB T3	-0.011	0.040	0.780	-0.014
CB T1 → Loneliness T2	0.036	0.066	0.587	0.026
CB T2 → Loneliness T3	0.036	0.066	0.587	0.026
**Within-person correlations**				
Loneliness T1 ←→ CB T1	0.000	0.021	0.992	0.001
Loneliness T2 ←→ CB T2	0.010	0.022	0.649	0.024
Loneliness T3 ←→ CB T3	**0.050**	**0.018**	**0.006**	**0.116**
**Between-person correlations**				
Loneliness ←→ CB	**0.127**	**0.025**	**<.001**	**0.246**
**Model fit**				
	$X^2(5) = 4.21$, $p = .520$, RMSEA < .01, SRMR = .01, CFI = .99			

Table A8

Results of the Constrained Three-wave RI-CLPM Between Specific Conspiracy Beliefs, Loneliness, and Meeting People face-to-face Controlling for Gender, Age, and Education

	B	SE	*p*	β
**Autoregressions**				
Loneliness T1 → Loneliness T2	**0.442**	**0.053**	**< .001**	**. 334**
Loneliness T2 → Loneliness T3	**0.442**	**0.053**	**< .001**	**.433**
CB T1 → CB T2	**0.264**	**0.059**	**< .001**	**.232**
CB T2 → CB T3	**0.264**	**0.059**	**< .001**	**.280**
F2F Contact T1 → F2F Contact T2	**0.273**	**0.078**	**< .001**	**.263**
F2F Contact T2 → F2F Contact T3	**0.273**	**0.078**	**< .001**	**.232**
**Cross-Lagged Effects**				
Loneliness T1 → CB T2	0.013	0.036	.718	.013
Loneliness T2 → CB T3	0.013	0.036	.718	.018
Loneliness T1 → F2F Contact T2	-0.018	0.047	.708	-.017
Loneliness T2 → F2F Contact T3	-0.018	0.047	.708	-.019
CB T1 → Loneliness T2	0.031	0.057	.590	.020
CB T2 → Loneliness T3	0.031	0.057	.590	.023
CB T1 → F2F Contact T2	0.047	0.060	.435	.039
CB T2 → F2F Contact T3	0.047	0.060	.435	.038
F2F Contact T1 → Loneliness T2	-0.062	0.054	.249	-.049
F2F Contact T2 → Loneliness T3	-0.062	0.054	.249	-.049
F2F Contact T1 → CB T2	0.012	0.045	.789	.013
F2F Contact T2 → CB T3	0.012	0.045	.789	.014
**Within-person correlations**				
CB T1 ←→ Loneliness T1	-0.021	0.022	.340	-.065
CB T1 ←→ F2F Contact T1	0.021	0.022	.338	.063
Loneliness T1 ←→ F2F Contact T1	-0.009	0.029	.756	-.023
CB T2 ←→ Loneliness T2	0.041	0.021	.050	.091
CB T2 ←→ F2F Contact T2	0.029	0.020	.152	.078
Loneliness T2 ←→ F2F Contact T2	-0.000	0.023	.990	-.001
CB T3 ←→ Loneliness T3	**0.043**	**0.017**	**.013**	**.104**
CB T3 ←→ F2F Contact T3	0.033	0.017	.057	.080
Loneliness T3 ←→ F2F Contact T3	0.030	0.021	.160	.053
**Between-person correlations**				
Loneliness ←→ CB	**0.104**	**0.025**	**< .001**	**.237**
Loneliness ←→ F2F Contact	**0.079**	**0.030**	**.009**	**.214**
F2F Contact ←→ CB	**0.078**	**0.024**	**.001**	**.218**
**Model fit**				
	$X^2(12) = 15.65$, $p = .208$, RMSEA = .01, SRMR = .01, CFI = .99			

Table A9

Results of the Constrained Three-wave RI-CLPM Between Specific Conspiracy Beliefs, Loneliness, and Perceived Health Risk Controlling for Gender, Age, and Education

	B	SE	*p*	β
**Autoregressions**				
Loneliness T1 → Loneliness T2	**0.449**	**0.053**	**< .001**	**.339**
Loneliness T2 → Loneliness T3	**0.449**	**0.053**	**< .001**	**.440**
CB T1 → CB T2	**0.258**	**0.059**	**< .001**	**.229**
CB T2 → CB T3	**0.258**	**0.059**	**< .001**	**.274**
Health Risk T1 → Health Risk T2	0.134	0.085	.116	.139
Health Risk T2 → Health Risk T3	0.134	0.085	.116	.121
**Cross-Lagged Effects**				
Loneliness T1 → CB T2	0.013	0.036	.729	.013
Loneliness T2 → CB T3	0.013	0.036	.729	.018
Loneliness T1 → Health Risk T2	-0.006	0.056	.922	-.005
Loneliness T2 → Health Risk T3	-0.006	0.056	.922	-.006
CB T1 → Loneliness T2	0.026	0.057	.646	.017
CB T2 → Loneliness T3	0.026	0.057	.646	.019
CB T1 → Health Risk T2	-0.058	0.074	.431	-.046
CB T2 → Health Risk T3	-0.058	0.074	.431	-.047
Health Risk T1 → Loneliness T2	-0.039	0.049	.424	-.035
Health Risk T2 → Loneliness T3	-0.039	0.049	.424	-.033
Health Risk T1 → CB T2	-0.053	0.042	.206	-.062
Health Risk T2 → CB T3	-0.053	0.042	.206	-.063
**Within-person correlations**				
CB T1 ←→ Loneliness T1	-0.021	0.023	.355	-.063
CB T1 ←→ Health Risk T1	-0.036	0.022	.094	-.094
Loneliness T1 ←→ Health Risk T1	-0.008	0.027	.769	-.018
CB T2 ←→ Loneliness T2	0.040	0.021	.056	.089
CB T2 ←→ Health Risk T2	-0.038	0.027	.160	-.093
Loneliness T2 ←→ Health Risk T2	-0.002	0.029	.936	-.004
CB T3 ←→ Loneliness T3	**0.041**	**0.017**	**.016**	**.100**
CB T3 ←→ Health Risk T3	**0.038**	**0.018**	**.037**	**.091**
Loneliness T3 ←→ Health Risk T3	-0.035	0.023	.123	-.062
**Between-person correlations**				
RI Loneliness ←→ RI CB	**0.105**	**0.025**	**< .001**	**.242**
RI Loneliness ←→ RI Health Risk	**-0.094**	**0.032**	**.003**	**-.182**
RI Health Risk ←→ RI CB	0.008	0.026	.752	.016
**Model fit**				
		$X^2(12) = 21.36, p = .045$, RMSEA = .02, SRMR = .01, CFI = .99		

References

Chen, F. F. (2007). Sensitivity of goodness of fit indexes to lack of measurement invariance. *Structural Equation Modeling: A Multidisciplinary Journal*, *14*(3), 464–504. https://doi.org/10.1080/10705510701301834

Cheung, G. W., & Rensvold, R. B. (2002). Evaluating Goodness-of-Fit Indexes for testing measurement invariance. *Structural Equation Modeling: A Multidisciplinary Journal*, *9*(2), 233–255. https://doi.org/10.1207/s15328007sem0902_5

Mackinnon, S. P., Curtis, R., & O'Connor, R. M. (2022). Tutorial in longitudinal measurement invariance and cross-lagged panel models using Lavaan. *Meta-psychology*, *6*. https://doi.org/10.15626/mp.2020.2595

Mulder, J. D. (2022). Power analysis for the random intercept cross-lagged panel model using the powRICLPM R-Package. *Structural Equation Modeling: A Multidisciplinary Journal*, *30*(4), 645–658. https://doi.org/10.1080/10705511.2022.2122467

Pummerer, L., Böhm, R., Lilleholt, L., Winter, K., Zettler, I., & Sassenberg, K. (2021). Conspiracy theories and their societal effects during the COVID-19 pandemic. *Social Psychological and Personality Science*, *13*(1), 49–59. https://doi.org/10.1177/19485506211000217

Rutjens, B. T., & van der Lee, R. (2020). Spiritual skepticism? Heterogeneous science skepticism in the Netherlands. *Public Understanding of Science (Bristol, England)*, *29*(3), 335–352. https://doi.org/10.1177/0963662520908534

van De Schoot, R., Lugtig, P., & Hox, J. J. (2012). A checklist for testing measurement invariance. *European Journal of Developmental Psychology*, *9*(4), 486–492. https://doi.org/10.1080/17405629.2012.686740
